# CGG expansion in *NOTCH2NLC* is associated with oculopharyngodistal myopathy with neurological manifestations

**DOI:** 10.1186/s40478-020-01084-4

**Published:** 2020-11-25

**Authors:** Masashi Ogasawara, Aritoshi Iida, Theerawat Kumutpongpanich, Ayami Ozaki, Yasushi Oya, Hirofumi Konishi, Akinori Nakamura, Ryuta Abe, Hiroshi Takai, Ritsuko Hanajima, Hiroshi Doi, Fumiaki Tanaka, Hisayoshi Nakamura, Ikuya Nonaka, Zhaoxia Wang, Shinichiro Hayashi, Satoru Noguchi, Ichizo Nishino

**Affiliations:** 1 Department of Neuromuscular Research, National Institute of Neuroscience, National Center of Neurology and Psychiatry (NCNP), 4-1-1 Ogawahigashi, Kodaira, Tokyo 187-8502 Japan; 2 Medical Genome Center, NCNP, Kodaira, Tokyo Japan; 3 Institute of Medical Genetics, Tokyo Women’s Medical University, Tokyo, Japan; 4 Department of Neurology, National Center Hospital, National Center of Neurology and Psychiatry, Tokyo, Japan; 5 Department of Neurology, Toyama University Hospital, Toyama, Japan; 6 Department of Clinical Research, National Hospital Organization Matsumoto Medical Center, Matsumoto, Japan; 7 Department of Neurology and Rheumatology, Shinshu University School of Medicine, Matsumoto, Japan; 8 Department of Neurology, Matsue City Hospital, Matsue, Japan; 9 Division of Neurology, Department of Brain and Neurosciences, Faculty of Medicine, Tottori University, Yonago, Japan; 10 Department of Neurology and Stroke Medicine, Yokohama City University Graduate School of Medicine, Fukuura, Kanazawa-ku, Yokohama, Japan; 11 Department of Neurology, Peking University First Hospital, Beijing, China

**Keywords:** Oculopharyngodistal myopathy, *NOTCH2NLC*, CGG repeat expansion, Neuronal intranuclear inclusion disease, Oculopharyngeal muscular dystrophy

## Abstract

Oculopharyngodistal myopathy (OPDM) is a rare hereditary muscle disease characterized by progressive distal limb weakness, ptosis, ophthalmoplegia, bulbar muscle weakness and rimmed vacuoles on muscle biopsy. Recently, CGG repeat expansions in the noncoding regions of two genes, *LRP12* and *GIPC1*, have been reported to be causative for OPDM. Furthermore, neuronal intranuclear inclusion disease (NIID) has been recently reported to be caused by CGG repeat expansions in *NOTCH2NLC*. We aimed to identify and to clinicopathologically characterize patients with OPDM who have CGG repeat expansions in *NOTCH2NLC* (OPDM_NOTCH2NLC). Note that 211 patients from 201 families, who were clinically or clinicopathologically diagnosed with OPDM or oculopharyngeal muscular dystrophy, were screened for CGG expansions in *NOTCH2NLC* by repeat primed-PCR. Clinical information and muscle pathology slides of identified patients with OPDM_NOTCH2NLC were re-reviewed. Intra-myonuclear inclusions were evaluated using immunohistochemistry and electron microscopy (EM). Seven Japanese OPDM patients had CGG repeat expansions in *NOTCH2NLC*. All seven patients clinically demonstrated ptosis, ophthalmoplegia, dysarthria and muscle weakness; they myopathologically had intra-myonuclear inclusions stained with anti-poly-ubiquitinated proteins, anti-SUMO1 and anti-p62 antibodies, which were diagnostic of NIID (typically on skin biopsy), in addition to rimmed vacuoles. The sample for EM was available only from one patient, which demonstrated intranuclear inclusions of 12.6 ± 1.6 nm in diameter. We identified seven patients with OPDM_NOTCH2NLC. Our patients had various additional central and/or peripheral nervous system involvement, although all were clinicopathologically compatible; thus, they were diagnosed as having OPDM and expanding a phenotype of the neuromyodegenerative disease caused by CGG repeat expansions in *NOTCH2NLC*.

## Introduction

Oculopharyngodistal myopathy (OPDM) is a rare adult-onset hereditary muscle disease clinically characterized by progressive ocular, pharyngeal, and distal limb muscle involvement and pathologically by rimmed vacuoles in muscle fibers [11, 20]. To date, two causative genes, *LRP12* and *GIPC1*, were identified for OPDM (thereby, we refer to them as OPDM_LRP12 and OPDM_GIPC1, respectively). In both diseases, CGG repeat expansions in the noncoding regions of the corresponding genes were believed to be the cause, although the pathogenesis remained largely unclear [2, 6]. Recently, CGG repeat expansions in the noncoding region of *NOTCH2NLC* were reported as causative for neuronal intranuclear inclusion disease (NIID), a slowly progressive neurodegenerative disorder with eosinophilic intranuclear inclusions in the central and peripheral nervous systems and various organs [6, 13, 19]. Interestingly, certain patients with NIID manifested muscle weakness, dysarthria and dysphagia, and not fully but partially mimicking OPDM. Nevertheless, a diagnosis of OPDM has never been made in these patients most possibly because ocular symptoms, an essential feature of OPDM, were lacking [9, 14]. However, patients with OPDM were rarely reported to have accompanying sensorineural hearing loss and demyelinating neuropathy [3]. The presence of such patients with NIID and OPDM raised a possibility that OPDM in certain patients, particularly those with additional neurological manifestations, could be caused by the same pathogenic mechanism as NIID. Therefore, we evaluated CGG expansions in *NOTCH2NLC* in patients who were suspected to have OPDM.

## Materials

### Inclusion criteria of patients

The National Center of Neurology and Psychiatry (NCNP) functions as a referral center for muscle diseases in Japan, thus providing pathological and genetic diagnoses. Among the samples that were sent to the NCNP for diagnostic purposes from 1978 to 2020, we included 211 Japanese patients from 201 families who were clinically or clinicopathologically diagnosed with OPDM or oculopharyngeal muscular dystrophy (OPMD) based on a combination of at least two of ptosis, bulbar symptoms, or rimmed vacuoles on muscle biopsy but did not have the GCN repeat expansion in the polyadenylate binding protein nuclear 1 (*PABPN1*) gene, the causative factor for OPMD [1].

## Methods

### Genetic analysis

Note that 211 patients were screened for CGG expansions in *NOTCH2NLC* by repeat primed-PCR (RP-PCR), and the CGG repeat length in patients who had expanded CGG repeats was determined by fragment analysis and/or Southern blotting, as previously described [6, 13]. The CGG repeats in *LRP12* and *GIPC1* were also evaluated by RP-PCR, fragment analysis, and/or Southern blotting [2, 6].

### Muscle histology

We re-reviewed the muscle pathology slides of a battery of histochemical stains that were prepared at the time of diagnosis. Immunohistochemical analysis of 8-µm-thick serial frozen sections was performed using the following primary antibodies: anti-poly-ubiquitinated proteins (FK1, BML-PW8805, 1:100; Enzo Life Sciences), anti-SUMO-1 (D-11:sc-5308, 1:50; Santa Cruz Biotechnology), anti-phospho-p62/SQSTM1 (Ser351) (PM074, 1:500; Medical & Biological Laboratories), and anti-caveolin-3 (sc7665, 1:200; Santa Cruz Biotechnology). After incubation with primary antibodies, the sections were incubated with DAPI (Cellstain DAPI, 1:1000; Fujifilm) and Alexa Fluor 488-, 568-, and 647-conjugated secondary antibodies (1:600; Invitrogen). Images were obtained using Keyence CCD camera (Keyence, Osaka, Japan). For evaluating regenerating fibers, we performed immunohistochemical analysis of serial frozen sections using neonatal myosin heavy chain (nMHC, NCL-MHCn, 1:20; Leica Biosystems Newcastle). We analyzed the frequency of regenerating fibers, fibers with internal nuclei, fibers with rimmed vacuoles, small angular fibers, and type 2C fibers in 200 randomly selected muscle fibers in each patient. Moreover, we analyzed the frequency of myonuclei positive with anti-p62, anti-poly-ubiquitinated protein, and anti-SUMO antibodies in 200 randomly selected myonuclei. Glutaraldehyde-fixed muscle sample for EM was available only from patient 2. On EM, we measured the diameter of 75 randomly selected intra-myonuclear filaments, which were straight, non-overlapped and well-demarcated.

### Skin histology

Skin biopsy samples were available from two patients (patients 1 and 7). Serial sections that had a thickness of 4 µm were fixed in formalin and stained by hematoxylin and eosin (H&E) and anti-p62/SQSTM1 antibody (sc-28359, 1;200, Santa Cruz Biotechnology).

## Results

**Fig. 1:**
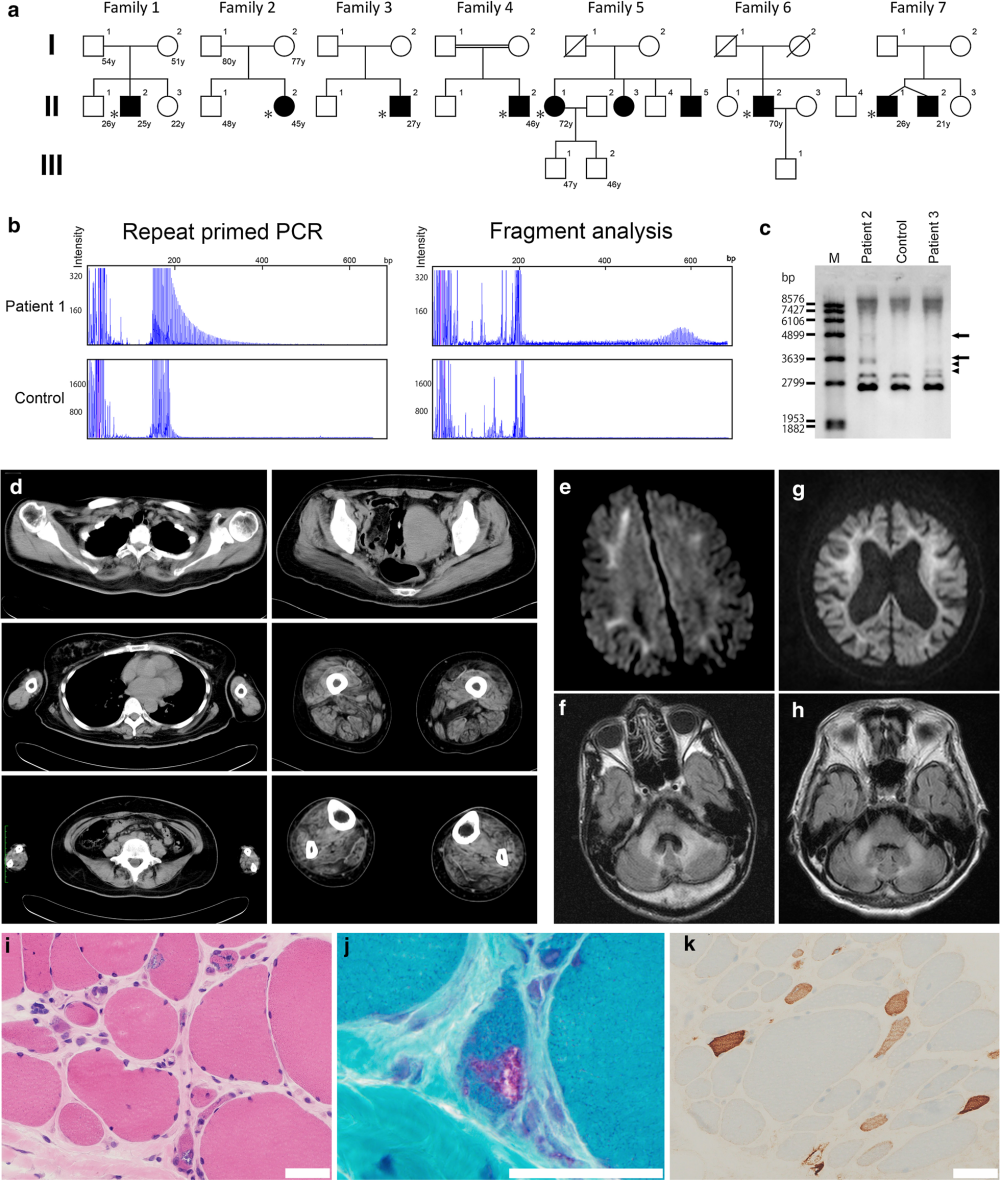
Clinical information of patients with CGG expansion in *NOTCH2NLC*. **a** Family trees of seven families. White circles and squares show unaffected indivisuals. Black circles and squares show patients. * shows probands. **b** Repeat primed PCR and fragment analysis for patient 1 and control. **c** Southern blotting analysis for CGG expansion in *NOTCH2NLC*. The arrows and arrowheads indicate the expanded alleles in patients 2 and 3, respectively. M, DNA marker. **d** Imaging of muscle CT from patient 2 shows asymmetric muscle atrophy. Right gluteus maximus, left vastus lateralis, adductor magnus, and rectus femoris are severely affected. **e** DWI of patient 1. **f** FLAIR image of patient 1. **g** DWI of patient 5. **h** FLAIR image of patient 5. **i** Marked fiber size variation, moderate endomysial fibrosis, and fibers with internal nuclei are seen on H&E. **j** Rimmed vacuole is present in a muscle fiber on mGT. **k** Some myofibers express neonatal myosin heavy chain. **i**–**k** Scale bars denote 50 µm

We screened 211 patients with a clinical or clinicopathological diagnosis of OPDM or OPMD and identified seven unrelated patients with OPDM with CGG expansions in *NOTCH2NLC* (OPDM_NOTCH2NLC) using RP-PCR. Among them, five were sporadic while two had affected siblings (Fig. 1a). Repeat units were determined by fragment analysis in five patients (patients 1, 4, 5, 6, and 7) and by Southern blotting in two patients (patients 2 and 3) (Fig. 1a–c). All patients had > 100 repeat expansions, and patient 2 had two long CGG expansions, the longer one being approximately 674 repeats (Fig. 1a–c and Table 1). None of the seven patients had expanded CGG repeats in *LRP12* and *GIPC1*.

**Table 1:** Clinical symptoms of patients with CGG expansion in *NOTCH2NLC*

Patient ID	1	2	3	4	5	6	7
Sex	F	F	M	M	F	M	M
Onset age	1 year	27 years	17 years	44 years	55 years	68 years	20 years
Repeat size	139	217,674	83, 184	116	135	132	128
Initial symptom	Dev	MW	MW, RPD	Ptosis	RPD	MW	PD
Ptosis	+	+	+	+	+	+	+
Ophthalmoplegia	+	+	+	+	+	+	+
Dysphagia	+	+	+	-	-	+	+
Dysarthria	+	+	+	+	+	+	+
Facial weakness	+	+	+	-	-	+	+
Limb muscle weakness	+	+	+	+	+	+	+
Distal/proximal/both	D (TA, Pe)	D > P	D > P	D (TA) > P	PU	D = P	D > P
Other muscle weakness	Neck	Neck	Neck	-	-	-	-
Muscle atrophy	Diffuse	Distal	Diffuse	Distal	-	-	Diffuse
Deep tendon reflex	↓↓	↓↓	↓	↓	↓	↓↓	↓↓
Sensory disturbance	-	-	-	+	+	+	-
Cerebellar symptoms Tremor	Ataxia	-	-	Tremor	Ataxia	NA	Ataxia
Eye abnormality	VD	RP	RPD	Mio, Pho	RPD	Cataract	-
Ear abnormality	Co	SHL	NA	+	+	+	-
HDSR	20	30	29	28	6	20	NA
Leukoencephalopathy	+	-	NA	NA	+	-	+
Neuropathy confirmed by NCV	+	NA	-	+	NA	+	+
ECG, UCG	-	Long QT	-	-	-	AV block, LVH	-
CSF cells per µl	2	NA	NA	2	1	1	4
CSF protein, mg/dl	60	NA	NA	110	157	77	59
CK IU/L	203	654	1475	1886	63	735	436
Lactate mg/dl	19.6	14.0	17.3	NA	21.6	5.3	9.7

*Co* conductive deafness, *Dev* developmental delay, *DL* distal lower limb, *DU* distal upper limb, *HDSR* Hasegawa dementia rating scale-revised, *LVH* left ventricular hypertrophy; *Mio* miosis, *MW* muscle weakness, *N* neck weakness, *NA* not available, *PD* panic disorder, *Pe* peroneal, *Pho* photophobia, *PU* proximal upper limb, *RP* retinal pigmentation, *RPD* retinal pigmentary degeneration, *SHL* sensory hearing loss, *TA* tibialis anterior, *VD* visual disturbance

Table 1 summarizes the clinical information of the seven patients with CGG expansions in *NOTCH2NLC*. The age at onset extensively varied from infancy to 67 years. All seven patients had ptosis, ophthalmoplegia, dysarthria, limb muscle weakness, and decreased deep tendon reflex. Dysphagia and facial muscle weakness were detected in five (71%) patients. Five (71%) patients had predominantly distal limb muscle weakness and muscle atrophy, whereas one (14%) patient had proximal upper limb muscle weakness. In addition to these clinical manifestations typical of OPDM, all patients had central or peripheral nervous system abnormalities such as leukoencephalopathy, retinal pigmentary degeneration, ataxia, tremor, deafness, peripheral neuropathy, and increased CSF protein levels. The CK level was moderately increased (436–1886 U/L) in five (71%) patients. Patient 6 had left ventricular hypertrophy with first-degree atrioventricular block, whereas patient 2 had long QT syndrome.

Muscle CT data were available for three patients. One of three patients had asymmetric muscle involvement with fat replacement markedly in the left adductor magnus and moderately in the right gluteus maximus, left vastus lateralis, and left rectus femoris (Fig. 1d). The other two patients showed muscle atrophy in calf muscles. Brain MR examinations were available in five patients (Fig. 1e–h). High-intensity signals were observed in the middle cerebellar peduncles on fluid-attenuated inversion recovery (FLAIR) images in three patients (patients 1, 5, and 7). Furthermore, high signals on FLAIR were observed in the medial part of the cerebellar hemisphere right beside the vermis and cerebral white matter in two patients (patients 1 and 5). High signals were noted along the corticomedullary junction on diffusion-weighted imaging (DWI) in two patients (patients 1 and 5). Moreover, moderate to marked ventricular enlargement was observed in three of five patients.

**Fig. 2:**
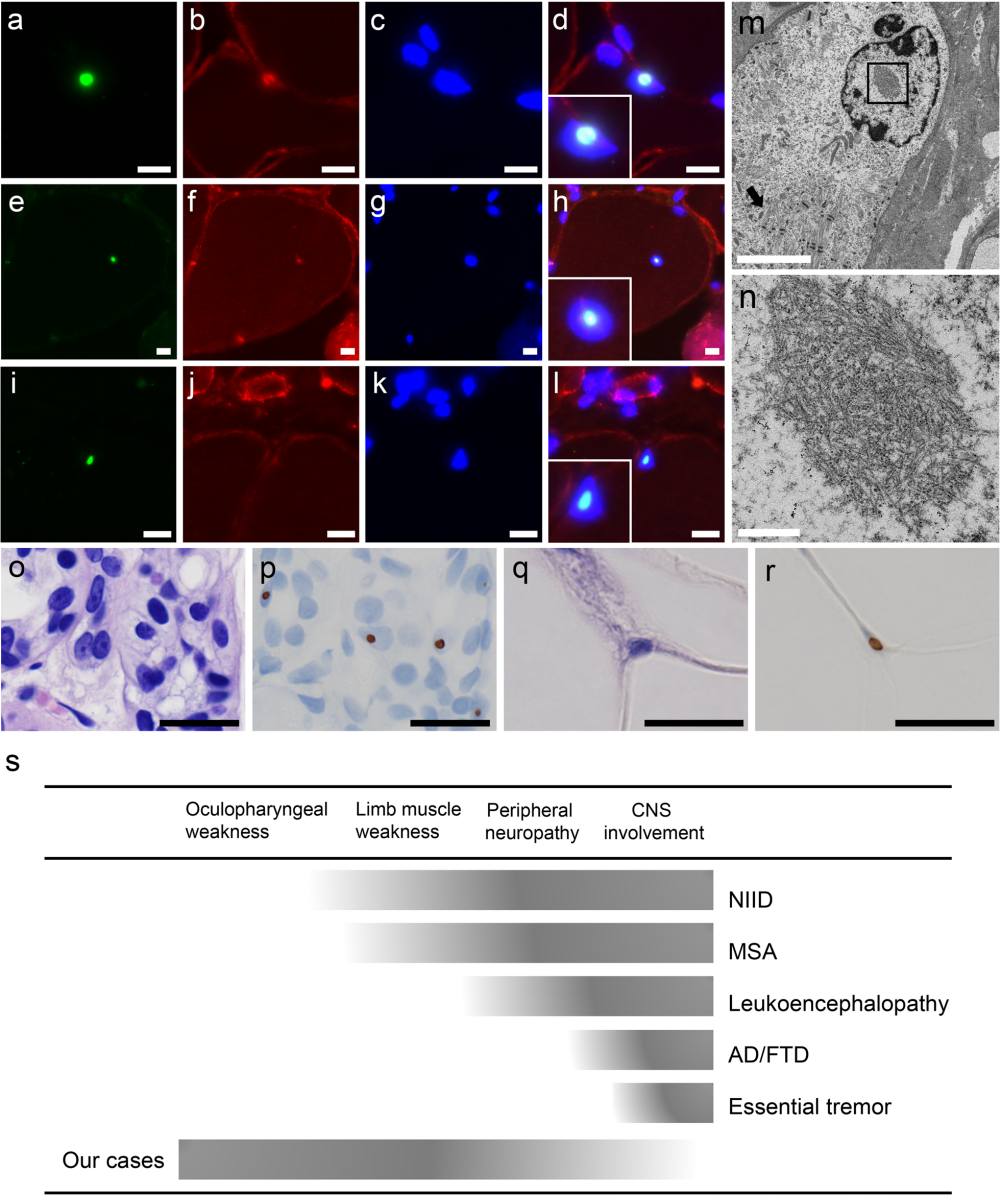
Pathology, revealed by electron microscopy and disease spectrum. Anti-poly-ubiquitinated protein antibody (**a**), anti-caveolin-3 antibody (**b**, **f**, **j**), DAPI (**c**, **g**, **k**), anti-SUMO-1 antibody (**e**), and anti-p62 (**i**) are stained. **d**, **h**, **l** show merged immunohistochemistry. **a**–**l** Scale bars denote 10 µm. On EM, a longitudinal section shows tubulofilamentous inclusions within the myonuclei together with markedly disorganized myofibrils (arrow) in the surrounding area (**m**, **n**). **m** Scale bar denotes 5 µm. **n** Scale bar shows 500 nm. **o**, **q** HE, **p**, **r** p62 are stained on serial sections of the skin sample in patient 1. Intranuclear inclusions with p62-positive in sweat gland cells (**p**) and adipocytes (**r**) are seen. **o**–**r** Scale bars denote 20 µm. **s** Disease spectrum caused by CGG expansion in *NOTCH2NLC*

On muscle histology, all patients had fibers with rimmed vacuoles and small angular fibers (Fig. 1i, j, and Table 2). The variation in fiber size was moderate to marked in six patients and mild in one patient. Six patients had regenerating fibers, and one patient had no necrotic or regenerating fibers (Fig. 1k and Table 2). Only one patient (patient 3) had moderate endomysial fibrosis, whereas the other patients had no or only minimal endomysial fibrosis (Fig. 1i and Table 2). Intra-myonuclear inclusions, primarily located in the center of the myonuclei, were detected using anti-poly-ubiquitinated protein, anti-SUMO1, and anti-phospho-p62/SQSTM1 antibodies in all patients (Fig. 2a–l). EM analysis of the muscle from patient 2 revealed tubulofilamentous inclusions without limiting membrane but with low-electron density halo around the nucleus, located in the center of the myonuclei (Fig. 2m,n). The diameter of the 75 randomly chosen filaments was 12.6 ± 1.6 nm (M ± SD).

**Table 2:** Pathological findings of patients with CGG expansion in *NOTCH2NLC*

Patient ID	1	2	3	4	5	6	7
Sex	F	F	M	M	F	M	M
Age at muscle biopsy	25 years	45 years	27 years	46 years	72 years	70 years	26 years
Fiber size variation	Moderate	Marked	Marked	Mild	Moderate	Moderate	Moderate
Regenerating fibers	2%	2%	16%	1%	-	0.5%	5%
Small angular fibers	4%	10.5%	5%	1%	8.5%	8%	10%
Endomysial fibrosis	±	±	++	±	-	±	±
Fibers with internal nuclei	2%	5.5%	4.5%	19%	1%	17.5%	3%
Fibers with rimmed vacuoles	1.5%	3.5%	4%	0.5%	0.5%	2%	2.5%
Type 2C fibers	1.5%	1%	2%	1.5%	1.5%	2%	3.5%
Intra-myonuclear p62 positive	3.5%	2.5%	7%	2.5%	1%	3%	2%
Intra-myonuclear ubiquitin positive	2%	0.5%	2.5%	1.5%	2.5%	0.5%	2.5%
Intra-myonuclear SUMO1 positive	2.5%	0.5%	2.5%	2.5%	2.5%	1%	2%

*M* male, *F* female

Skin biopsies from patients 1 and 7 showed p62-positive intranuclear inclusions (Fig. 2o–r).

## Discussion

NIID is a slowly progressive neurodegenerative disorder that is pathologically characterized by eosinophilic hyaline intranuclear inclusions in the central and peripheral nervous systems, as well as in the visceral organs and skin. This disorder has been considered to be a heterogeneous disease because of the highly variable clinical manifestations [14]. Recent studies reported noncoding CGG repeat expansions in *NOTCH2NLC* as the causative factor for NIID [6, 13, 19]. Subsequently, various diseases have been associated with CGG repeat expansions in *NOTCH2NLC*, including multiple system atrophy (MSA), leukoencephalopathy, Alzheimer’s disease and frontotemporal dementia (AD/FTD), tremor, and retinal dystrophy, suggesting that the spectrum of *NOTCH2NLC* diseases is, in fact, wide (Fig. 2s) [4, 5, 7, 10, 17].

Interestingly, certain patients with NIID demonstrate additional myopathic features such as limb muscle weakness, dysphagia, and dysarthria [9, 14]. However, neither ptosis/ophthalmoplegia, a clinical hallmark of OPDM, nor rimmed vacuole, a pathological hallmark of OPDM, has never been described in any histologically or genetically confirmed NIID patient [14, 18]. Not surprisingly, to our knowledge, no patients with NIID have ever been clinicopathologically diagnosed with OPDM and vice versa.

Although our patients with OPDM_NOTCH2NLC were clinicopathologically diagnosed with OPDM, they had additional clinical manifestations that were partially reminiscent of NIID, including leukoencephalopathy and retinal degeneration. Moreover, one and three patients had tremor and ataxia. MRI results in two of five patients revealed high-intensity signals in the middle cerebellar peduncles and in the paravermal area on FLAIR images and in the corticomedullary junction on DWI, similar to those in patients with NIID (Fig. 1e–h) [6, 12, 16].

The identification of patients with OPDM_NOTCH2NLC suggests that CGG expansions in *NOTCH2NLC* result in at least two different diseases, namely NIID and OPDM. Nevertheless, because our patients had peripheral and/or central nervous system involvement together with the clinicopathological features of OPDM, which fills the phenotypic gap between the two diseases, they are most likely in a broad phenotypic spectrum of a single neuromyodegenerative disease rather than in two distinct diseases (Fig. 2s). The identification of the intranuclear inclusions in skin biopsy from two patients with OPDM_NOTCH2NLC, which is a diagnostic finding of NIID [15], as well as supports this notion further.

A recent study reported that patients with the muscle subtype of NIID had longer CGG repeat expansions from 118 to 517 repeats than those with other NIID subtypes [19]. Indeed, the CGG repeats in our patients ranged from 116 to 674. Interestingly, the patient (patient 2) carrying 674 repeats, although with mosaicism, had milder phenotype than the patient (patient 1) carrying 139 repeats who had an early-onset and severe phenotype, suggesting that there was no apparent correlation between the size of the CGG repeats and the clinical symptoms in OPDM_NOTCH2NLC.

Diagnostically, the presence of intranuclear inclusions stained with anti-poly-ubiquitinated protein, anti-SUMO1, and anti-p62 antibodies in the skin and other organs is pathognomonic of NIID [15]. In this study, we confirm the presence of essentially the same type of intranuclear inclusions in muscles, further suggesting that OPDM and NIID may be in the same spectrum with the identical degenerative process; although different organs, such as skeletal muscle, skin, and CNS, are affected in variable degrees. Interestingly, the average diameter of the intranuclear filaments was 12.6 nm on EM, which was similar to that observed in neuronal cells in patients with NIID (8–16 nm) [8, 21], suggesting that the underlying mechanism of the myodegeneration in OPDM_NOTCH2NLC and the neurodegeneration in NIID could be identical.

In terms of the pattern of muscle involvement on muscle imaging, one study described predominantly involved soleus and long head of the biceps femoris, relatively preserved rectus femoris, and asymmetric involvement pattern in OPDM (without genetic diagnosis) [22]. Another study reported markedly involved soleus in patients with OPDM_GIPC1 [2]. The results of muscle CT in the present three patients with OPDM_NOTCH2NLC are similar to previous reports [2, 22], suggesting that the myodegeneration mechanism for those three OPDM types may be similar.

In conclusion, our seven Japanese patients with OPDM_NOTCH2NLC exhibit distinct clinicopathological features, including the involvement of central and peripheral nervous systems. Our results widen the phenotypic spectrum of a neuromyodegenerative disease caused by CGG repeat expansions in *NOTCH2NLC*.

## Data Availability

The data supporting the results of this study are available from the corresponding author upon request.

## Abbreviations

OPMD: oculopharyngeal muscular dystrophy
OPDM: oculopharyngodistal myopathy
OPDM_NOTCH2NLC: OPDM patients who have CGG repeat expansions in *NOTCH2NLC*
NCNP: the National Center of Neurology and Psychiatry
NIID: neuronal intranuclear inclusion disease
PABPN1: polyadenylate binding protein nuclear 1
MSA: multiple system atrophy
AD/FTD: leukoencephalopathy, Alzheimer’s disease and frontotemporal dementia

## Clinical information

For patients with CGG expansions in *NOTCH2NLC*, clinical information that included imaging data of muscle and brain provided by the physicians at the time of muscle biopsy and/or genetic analysis was reviewed.

## References

[1] Brais B, Bouchard JP, Xie YG, Rochefort DL, Chretien N, Tome FM, Lafreniere RG, Rommens JM, Uyama E, Nohira O (1998). Short GCG expansions in the PABP2 gene cause oculopharyngeal muscular dystrophy. Nat Genet.

[2] Deng J, Yu J, Li P, Luan X, Cao L, Zhao J, Yu M, Zhang W, Lv H, Xie Z (2020). Expansion of GGC repeat in GIPC1 is associated with oculopharyngodistal myopathy. Am J Hum Genet.

[3] Durmus H, Laval SH, Deymeer F, Parman Y, Kiyan E, Gokyigiti M, Ertekin C, Ercan I, Solakoglu S, Karcagi V (2011). Oculopharyngodistal myopathy is a distinct entity: clinical and genetic features of 47 patients. Neurology.

[4] Fang P, Yu Y, Yao S, Chen S, Zhu M, Chen Y, Zou K, Wang L, Wang H, Xin L (2020). Repeat expansion scanning of the NOTCH2NLC gene in patients with multiple system atrophy. Ann Clin Transl Neurol.

[5] Hayashi T, Katagiri S, Mizobuchi K, Yoshitake K, Kameya S, Matsuura T, Iwata T, Nakano T (2020). Heterozygous GGC repeat expansion of NOTCH2NLC in a patient with neuronal intranuclear inclusion disease and progressive retinal dystrophy. Ophthal Genet.

[6] Ishiura H, Shibata S, Yoshimura J, Suzuki Y, Qu W, Doi K (2019). Noncoding CGG repeat expansions in neuronal intranuclear inclusion disease, oculopharyngodistal myopathy and an overlapping disease. Nat Genet.

[7] Jiao B, Zhou L, Zhou Y, Weng L, Liao X, Tian Y (2020). Identification of expanded repeats in NOTCH2NLC in neurodegenerative dementias. Neurobiol Aging.

[8] Malandrini A, Villanova M, Tripodi S, Palmeri S, Sicurelli F, Parrotta E (1998). Neuronal intranuclear inclusion disease: neuropathologic study of a case. Brain Dev.

[9] O’Sullivan JD, Hanagasi HA, Daniel SE, Tidswell P, Davies SW, Lees AJ (2000). Neuronal intranuclear inclusion disease and juvenile parkinsonism. Mov Disord.

[10] Okubo M, Doi H, Fukai R, Fujita A, Mitsuhashi S, Hashiguchi S (2019). GGC repeat expansion of NOTCH2NLC in adult patients with leukoencephalopathy. Ann Neurol.

[11] Satoyoshi E, Kinoshita M (1977). Oculopharyngodistal myopathy. Arch Neurol.

[12] Sone J, Kitagawa N, Sugawara E, Iguchi M, Nakamura R, Koike H (2014). Neuronal intranuclear inclusion disease cases with leukoencephalopathy diagnosed via skin biopsy. J Neurol Neurosurg Psychiatry.

[13] Sone J, Mitsuhashi S, Fujita A, Mizuguchi T, Hamanaka K, Mori K (2019). Long-read sequencing identifies GGC repeat expansions in NOTCH2NLC associated with neuronal intranuclear inclusion disease. Nat Genet.

[14] Sone J, Mori K, Inagaki T, Katsumata R, Takagi S, Yokoi S (2016). Clinicopathological features of adult-onset neuronal intranuclear inclusion disease. Brain.

[15] Sone J, Tanaka F, Koike H, Inukai A, Katsuno M, Yoshida M (2011). Skin biopsy is useful for the antemortem diagnosis of neuronal intranuclear inclusion disease. Neurology.

[16] Sugiyama A, Sato N, Kimura Y, Maekawa T, Enokizono M, Saito Y (2017). MR imaging features of the cerebellum in adult-onset neuronal intranuclear inclusion disease: 8 cases. AJNR Am J Neuroradiol.

[17] Sun QY, Xu Q, Tian Y, Hu ZM, Qin LX, Yang JX (2020). Expansion of GGC repeat in the human-specific NOTCH2NLC gene is associated with essential tremor. Brain.

[18] Tateishi J, Nagara H, Ohta M, Matsumoto T, Fukunaga H, Shida K (1984). Intranuclear inclusions in muscle, nervous tissue, and adrenal gland. Acta Neuropathol.

[19] Tian Y, Wang JL, Huang W, Zeng S, Jiao B, Liu Z (2019). Expansion of human-specific GGC repeat in neuronal intranuclear inclusion disease-related disorders. Am J Hum Genet.

[20] van der Sluijs BM, ter Laak HJ, Scheffer H, van der Maarel SM, van Engelen BG (2004). Autosomal recessive oculopharyngodistal myopathy: a distinct phenotypical, histological, and genetic entity. J Neurol Neurosurg Psychiatry.

[21] Woulfe JM (2007). Abnormalities of the nucleus and nuclear inclusions in neurodegenerative disease: a work in progress. Neuropathol Appl Neurobiol.

[22] Zhao J, Liu J, Xiao J, Du J, Que C, Shi X (2015). Clinical and muscle imaging findings in 14 mainland Chinese patients with oculopharyngodistal myopathy. PLoS ONE.

